# Impacted Molariform Distomolar Double Tooth: A Case Report

**DOI:** 10.7759/cureus.23780

**Published:** 2022-04-03

**Authors:** Karthik Rajaram Mohan, Ravikumar Pethagounder Thangavelu, Saramma Mathew fenn

**Affiliations:** 1 Oral Medicine and Radiology, Vinayaka Mission's Sankarachariyar Dental College, Vinayaka Mission's Research Foundation, Salem, IND

**Keywords:** tooth extraction, supernumerary teeth, fusion, distomolar, gemination

## Abstract

Double teeth, also called connated or cojoined teeth, are clinically present as two separate teeth united by dentin. It occurs due to the fusion of two individual tooth buds or the partial splitting of one into two. An accessory supernumerary fourth molar is called a distomolar or distodens. Usually, the distomolar has a small crown that can be conical, peg-shaped, or like a small premolar called molariform distomolar that occurs distal to the last molar. This case presents an impacted molariform distomolar with the fusion of crown and root in a 27-year-old female.

## Introduction

An accessory fourth molar is called a distomolar. It can be buccally or palatally placed in relation to the last molar tooth clinically or impacted within the alveolar bone. The distomolar, which resembles a premolar or molar-like tooth, is called a molariform distomolar tooth. Such impacted molariform distomolars are usually asymptomatic and are only discovered by routine radiographic examination. Though clinically asymptomatic, and impacted molariform distomolar can form a dentigerous cyst and requires removal. Recent advancements in maxillofacial imaging cone-beam CT help locate and visualize impacted molariform distomolars. Conventional intraoral periapical radiographs are futile, resembling an odontoma, and provide a diagnostic challenge to the dentist.

## Case presentation

**Figure 1:**
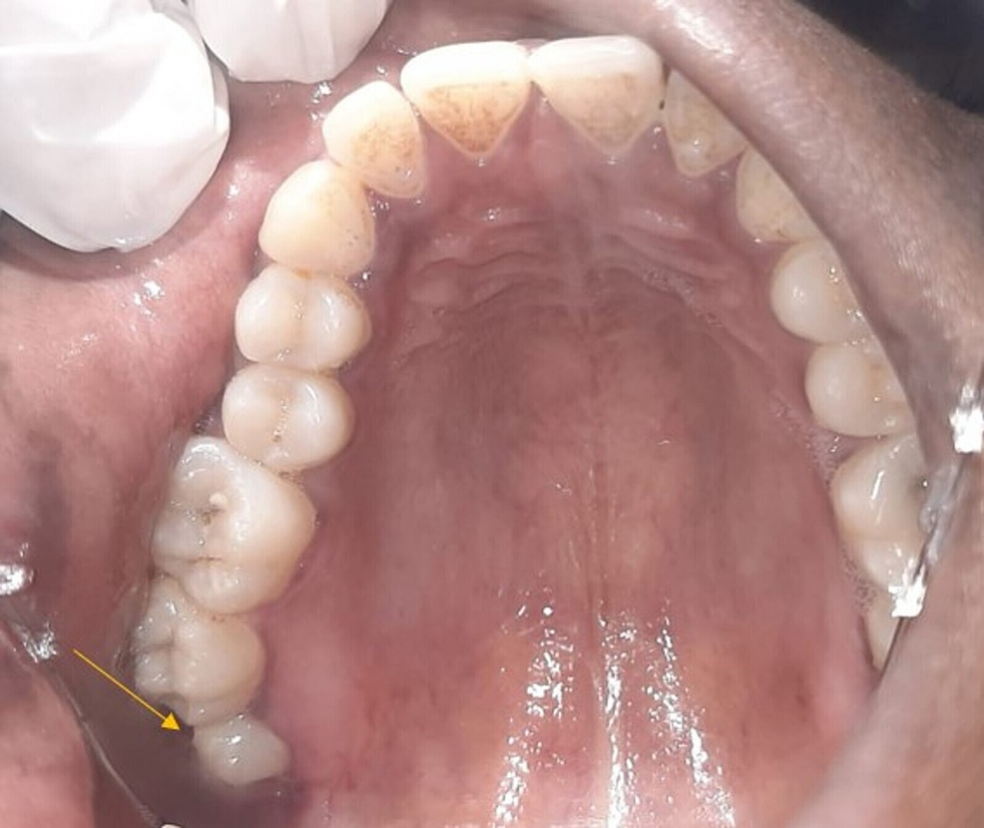
Intraoral clinical photograph revealed a carious maxillary right third molar.

A 27-year-old female reported to our Oral Medicine and Radiology Department with a chief complaint of pain in the decayed upper right back tooth region for the past 30 days. The patient initially experienced food lodgement that frequently occurred in the same tooth region due to cavitation caused by dental caries. On general examination, her vitals were stable. A single right submandibular lymph node was palpable, tender, firm in consistency, and movable. Intraoral examination revealed a carious right maxillary third molar (Figure 1).

**Figure 2:**
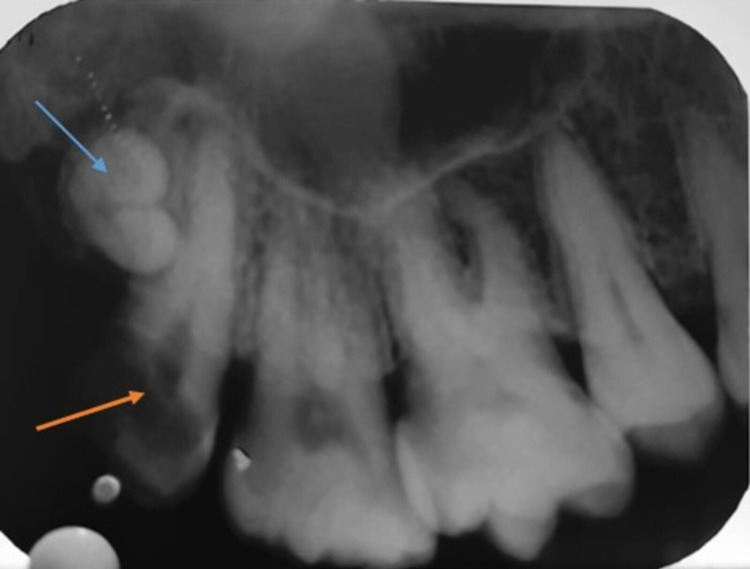
Intraoral Periapical Radiograph revealed dental caries approximating pulp in right maxillary third molar (orange arrow) and two radiopaque tooth-like radiopacity resembling odontoma (blue arrow)

An intraoral periapical radiograph revealed radiolucency involving the distoproximal crown portion approximating the pulp. Also, it revealed an additional radiopaque structure that resembles the crown of two small tooth-like structures distal to the carious right maxillary third molar tooth, suggestive of an odontoma (Figure 2).

**Figure 3:**
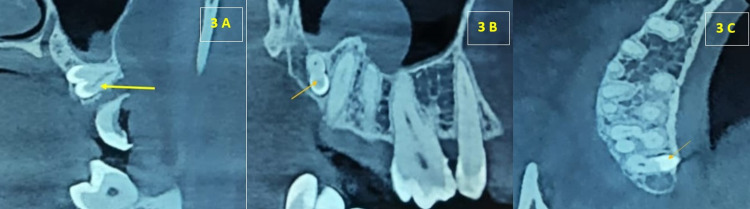
A. Coronal, Sagittal, Axial section cone-beam CT revealed fusion with a radiopaque structure in relation to palatal aspect of Maxillary Third Molar (orange arrow)

A cone-beam CT determined the exact location. The cone-beam CT coronal section revealed two crown-like radiopaque structures, one with an enamel cap and another with a central canal, attached to them within the maxillary alveolar bone. The Sagittal section of cone-beam CT revealed two fused crown and root-like radiopaque structures within the maxillary alveolar bone. The axial section of cone-beam CT revealed a small, dense, radiopaque structure on the palatal aspect of the root of the right maxillary third molar tooth (Figure 3A, 3B, 3C).

**Figure 4:**
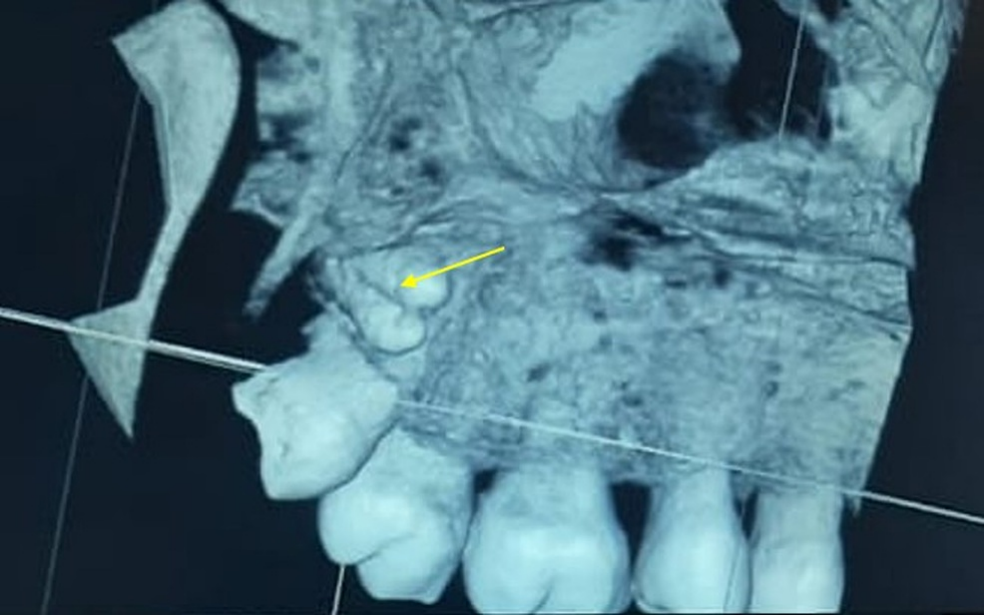
3D reconstructed CBCT revealed an impacted distomolar that resembled premolar toothlike (molariform) supernumerary teeth

The 3D- reconstructed cone-beam CT on the palatal aspect revealed a small tooth resembling the crown of a two-premolar tooth fused with a single root located palatal position (Figure 4).

The above radiographic findings allowed a final diagnosis of a palatally placed horizontally impacted molariform distomolar with fusion.

Treatment

**Figure 5:**
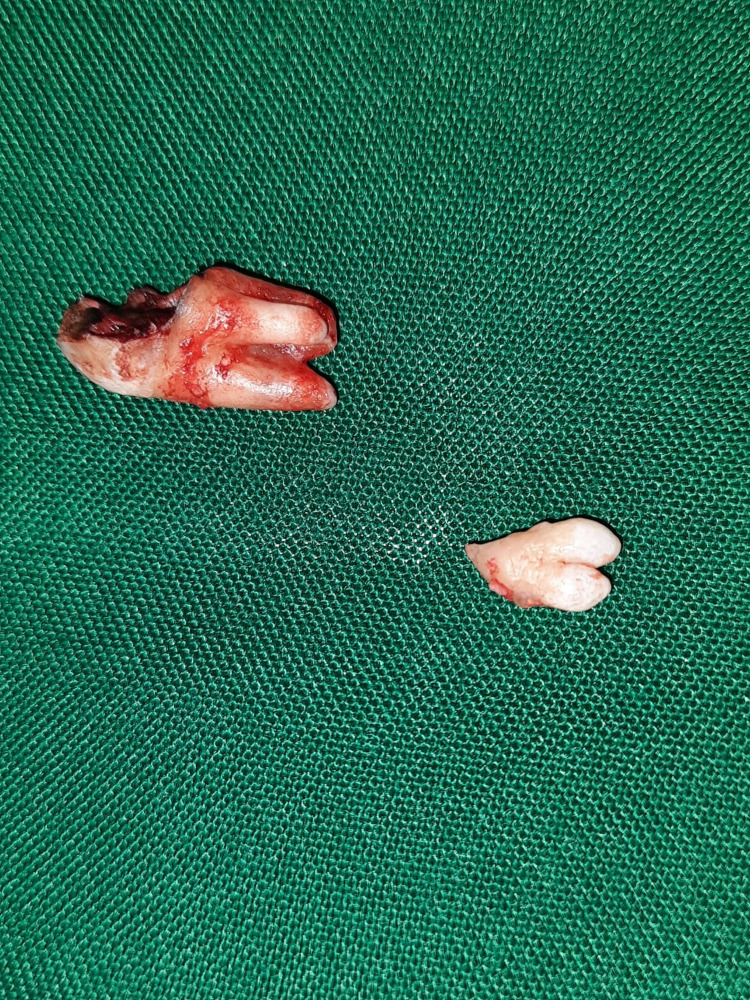
The extracted carious right maxillary third molar and impacted palatally placed molariform distomolar tooth

The decayed right maxillary third molar tooth was extracted with palatally placed impacted horizontally placed distomolar tooth under local anesthesia (Figure 5).

## Discussion

Supernumerary teeth are morphologically normal or abnormal and develop from excess dental lamina in the jaws during tooth formation in intrauterine life. Supernumerary teeth (ST) can form due to unknown developmental defects or underlying genetic reasons, and early extraction is recommended. A supernumerary tooth is called “Distomolar” or “Distodens” due to its location distally to the last molar tooth. An accessory fourth molar is called distomolar [1]. Mahto RK et al. reported the occurrence of posterior maxillary ST bilaterally [2]. Zhu F et al. Reported the occurrence of the supernumerary tooth in the mastoid bone [3]. Misirovs R et al. reported a supernumerary tooth in a nasopalatine canal, collapsing the septal cartilage [4]. A supernumerary tooth in the nasal cavity, called a nasal tooth, requires endoscopic assisted surgery [5]. Multiple impacted ST occurs in Nicolaides-Baratsier syndrome along with severe mental impairment with delay in speech, seizures, short stature, brachydactyly, and sparse hair [6]. Cleidocranial dysplasia is characterized by multiple supernumerary teeth, multiple missing teeth, and a partial or complete absence of clavicle. Gardners syndrome has multiple osteomas along with ST. Type I trichorhinophalangeal syndrome is an autosomal dominant disorder that includes ST in the maxillary molar region, skeletal anomalies with cone-shaped phalangeal epiphysis, a bulbous tip of the nose, long flat philtrum, protruding ears, thin maxillary vermilion border of the lip, and sparse scalp hairs [7].

An impacted distomolar can lead to the formation of a dentigerous cyst. Hence, removing such impacted distomolar helps prevent dentigerous cyst formation, which can later transform to squamous cell carcinoma. In addition, double teeth or connated teeth can have a pronounced groove on their surface, making them more caries prone, resulting in pain. During endodontic procedures like root canal therapy and dental extraction, the dental surgeon can encounter some clinical difficulty [8-10].

Kiso H et al. stated that in humans, the third dentition is generally apoptotically retarded, meaning that the teeth do not fully form. It was recently proposed that ST is the outcome of the rescue of the third dentition regression in humans [11]. Dental abnormalities develop due to genetic and environmental influences during the morphodifferentiation stage of odontogenesis, resulting in changes in the number, size, and shape of the tooth and the root [12].

## Conclusions

Impacted molariform distomolars are usually asymptomatic and are accidentally discovered by routine radiographic examination when a patient reports to radiology for other problems, such as pain from adjacent decayed tooth or food lodgement due to crowding or orthodontic treatment planning or facial asymmetry, caused by cystic degeneration of such impacted distomolar. A conventional radiographic technique like intraoral periapical radiograph of such impacted molariform distomolar can mimic an odontoma, giving rise to a diagnostic dilemma for the dentist for treatment. The advent of a recent imaging modality such as a cone-beam CT is helpful not only in the visualization of the exact location of such impacted molariform distomolar but also aid in treatment plans such as a surgical approach during dental extraction
